# Supplementary material for: Azolla filiculoides L. as a source of metal-tolerant microorganisms
Source: PLoS One. 2020 May 6;15(5):e0232699. doi: 10.1371/journal.pone.0232699 (PMC7202617; doi:10.1371/journal.pone.0232699)
Supplement: S1 Table — (DOCX) [file pone.0232699.s001.docx]

**S1 Table. The composition of ‘Other’ cluster of the representatives of Alphaproteobacteria (percentage of whole Proteobacteria).**

| **Genus** | **treatment** | | | | | | |
| --- | --- | --- | --- | --- | --- | --- | --- |
|  | **control** | **+Pb** | **+Cd** | **+Cr(VI)** | **+Ni** | **+Au** | **+Ag** |
| *Acidisoma* | 0 | 0.024 | 0.171 | 0.091 | 0.153 | 0.135 | 0.043 |
| *Acidocella* | 0 | 0.018 | 0 | 0.040 | 0 | 0 | 0 |
| *Aminobacter* | 0 | 0.024 | 0 | 0.022 | 0 | 0 | 0 |
| *Blastomonas* | 0 | 0 | 0.030 | 0 | 0 | 0 | 0 |
| *Bosea* | 0.048 | 0 | 0 | 0.011 | 0 | 0 | 0 |
| *Bradyrhizobium* | 0 | 0.030 | 0.038 | 0 | 0 | 0.025 | 0 |
| *Devosia* | 0 | 0 | 0.042 | 0.027 | 0 | 0 | 0 |
| *Dongia* | 0 | 0.012 | 0 | 0.046 | 0 | 0 | 0 |
| *Ensifer* | 0 | 0 | 0.080 | 0 | 0 | 0 | 0 |
| *Methylobacterium* | 0 | 0.027 | 0.030 | 0 | 0 | 0 | 0 |
| *Methylocystis* | 0.015 | 0 | 0.049 | 0.016 | 0 | 0 | 0 |
| *Novosphingobium* | 0 | 0.027 | 0.030 | 0.011 | 0 | 0 | 0 |
| *Paracoccus* | 0 | 0.012 | 0.053 | 0.035 | 0 | 0 | 0 |
| *Phenylobacterium* | 0 | 0.249 | 0 | 0 | 0 | 0 | 0 |
| *Rhodomicrobium* | 0 | 0.012 | 0 | 0 | 0 | 0 | 0 |
| *Rickettsia* | 0 | 0 | 0.095 | 0.042 | 0.092 | 0.044 | 0.595 |
| *Roseomonas* | 0 | 0 | 0.019 | 0.013 | 0 | 0 | 0 |
| *Shinella* | 0 | 0.033 | 0 | 0 | 0 | 0 | 0 |
| *Skermanella* | 0 | 0.012 | 0 | 0.009 | 0 | 0 | 0 |
| *Sphingobium* | 0.108 | 0.482 | 0 | 0 | 0 | 0 | 0 |
| *Sphingomonas* | 0 | 0.337 | 0.616 | 0.422 | 0.087 | 0.264 | 0.036 |
| *Sphingopyxis* | 0 | 0.110 | 0.030 | 0 | 0 | 0 | 0 |
