# Supplementary material for: Azolla filiculoides L. as a source of metal-tolerant microorganisms
Source: PLoS One. 2020 May 6;15(5):e0232699. doi: 10.1371/journal.pone.0232699 (PMC7202617; doi:10.1371/journal.pone.0232699)
Supplement: S2 Table — (DOCX) [file pone.0232699.s002.docx]

**S2 Table. The composition of ‘Other’ cluster of the representatives of Betaproteobacteria (percentage of whole Proteobacteria).**

| **Genus** | **treatment** | | | | | | |
| --- | --- | --- | --- | --- | --- | --- | --- |
|  | **control** | **+Pb** | **+Cd** | **+Cr(VI)** | **+Ni** | **+Au** | **+Ag** |
| *Achromobacter* | 0.054 | 0.024 | 0.015 | 0 | 0.014 | 0 | 0 |
| *Aquabacterium* | 0 | 0 | 0.179 | 0 | 0.012 | 0 | 0 |
| *Comamonas* | 0.246 | 0 | 0 | 0.015 | 0 | 0 | 0 |
| *Cupriavidus* | 0 | 0 | 0 | 0 | 0.026 | 0 | 0 |
| *Delftia* | 0.015 | 0 | 0 | 0 | 0 | 0 | 0 |
| *Hydrogenophaga* | 0 | 0.039 | 0 | 0 | 0 | 0 | 0 |
| *Massilia* | 0.008 | 0 | 0 | 0 | 0 | 0 | 0 |
| *Methylophilus* | 0 | 0.012 | 0 | 0 | 0 | 0.008 | 0 |
| *Oxalicibacterium* | 0 | 0 | 0 | 0.022 | 0 | 0 | 0 |
| *Pelomonas* | 0 | 0.101 | 0.445 | 0 | 0.026 | 0.022 | 0.036 |
| *Ralstonia* | 0 | 0.012 | 0 | 0 | 0 | 0 | 0 |
| *Thiobacillus* | 0 | 0.015 | 0 | 0 | 0 | 0 | 0 |
