# Supplementary material for: Azolla filiculoides L. as a source of metal-tolerant microorganisms
Source: PLoS One. 2020 May 6;15(5):e0232699. doi: 10.1371/journal.pone.0232699 (PMC7202617; doi:10.1371/journal.pone.0232699)
Supplement: S3 Table — (DOCX) [file pone.0232699.s003.docx]

**S3 Table. The composition of ‘Other’ cluster of the representatives of Deltaproteobacteria (percentage of whole Proteobacteria).**

| **Genus** | **treatment** | | | | | | |
| --- | --- | --- | --- | --- | --- | --- | --- |
|  | **control** | **+Pb** | **+Cd** | **+Cr(VI)** | **+Ni** | **+Au** | **+Ag** |
| *Labilithrix* | 0 | 0 | 0 | 0 | 0 | 0 | 0.010 |
| *Myxococcus* | 0 | 0 | 0.019 | 0 | 0 | 0 | 0 |
| *Nannocystis* | 0 | 0 | 0 | 0.009 | 0 | 0 | 0 |
| *Sandaracinus* | 0 | 0 | 0 | 0.007 | 0 | 0 | 0 |
| *Syntrophobacter* | 0 | 0 | 0.023 | 0 | 0 | 0 | 0 |
