# Supplementary material for: Azolla filiculoides L. as a source of metal-tolerant microorganisms
Source: PLoS One. 2020 May 6;15(5):e0232699. doi: 10.1371/journal.pone.0232699 (PMC7202617; doi:10.1371/journal.pone.0232699)
Supplement: S4 Table — (DOCX) [file pone.0232699.s004.docx]

**S4 Table. The composition of ‘Other’ cluster of the representatives of Gammaproteobacteria (percentage of whole Proteobacteria).**

| **Genus** | **treatment** | | | | | | |
| --- | --- | --- | --- | --- | --- | --- | --- |
|  | **control** | **+Pb** | **+Cd** | **+Cr(VI)** | **+Ni** | **+Au** | **+Ag** |
| *Aquicella* | 0 | 0 | 0 | 0.013 | 0 | 0 | 0 |
| *Azotobacter* | 0 | 0 | 0 | 0.024 | 0 | 0 | 0 |
| *Cardiobacterium* | 0 | 0 | 0 | 0.007 | 0 | 0 | 0 |
| *Enhydrobacter* | 0.015 | 0.015 | 0.015 | 0.018 | 0 | 0 | 0 |
| *Escherichia/Shigella* | 0 | 0.027 | 0 | 0 | 0 | 0 | 0 |
| *Haemophilus* | 0 | 0.012 | 0.034 | 0 | 0 | 0 | 0 |
| *Luteimonas* | 0 | 0 | 0.034 | 0 | 0 | 0 | 0 |
| *Lysobacter* | 0 | 0.015 | 0.027 | 0 | 0 | 0 | 0 |
| *Marinomonas* | 0 | 0.015 | 0 | 0 | 0 | 0 | 0 |
| *Methylomicrobium* | 0 | 0 | 0 | 0.006 | 0 | 0 | 0 |
| *Methylomonas* | 0 | 0 | 0 | 0.022 | 0 | 0 | 0 |
| *Nevskia* | 0 | 0 | 0 | 0 | 0.159 | 0 | 0.062 |
| *Pseudohongiella* | 0 | 0 | 0.034 | 0 | 0 | 0 | 0.010 |
| *Pseudoxanthomonas* | 0 | 0.204 | 0.015 | 0 | 0.060 | 0 | 0 |
| *Rhodanobacter* | 0 | 0.012 | 0.023 | 0 | 0 | 0 | 0 |
| *Serratia* | 0 | 0.012 | 0 | 0 | 0 | 0 | 0 |
| *Stenotrophomonas* | 0.874 | 0.068 | 0.023 | 0 | 0.014 | 0 | 0 |
| *Thermomonas* | 0 | 0 | 0.011 | 0 | 0 | 0 | 0 |
