# Supplementary material for: Azolla filiculoides L. as a source of metal-tolerant microorganisms
Source: PLoS One. 2020 May 6;15(5):e0232699. doi: 10.1371/journal.pone.0232699 (PMC7202617; doi:10.1371/journal.pone.0232699)
Supplement: S5 Table — (DOCX) [file pone.0232699.s005.docx]

**S5 Table. The relative abundance (%) of unidentified microorganisms (percentage of a given class of Proteobacteria) for each treatment and their assignment to corresponding family.**

| **Class** | **Family** | **treatment** | | | | | | |
| --- | --- | --- | --- | --- | --- | --- | --- | --- |
|  |  | **control** | **+Pb** | **+Cd** | **+Cr(VI)** | **+Ni** | **+Au** | **+Ag** |
| α-class | Acetobacteraceae | 0 | 0.036 | 0.046 | 0.121 | 0 | 0.006 | 0 |
|  | Beijerinckiaceae | 0 | 0 | 0 | 0.026 | 0.121 | 0.152 | 0 |
|  | Bradyrhizobiaceae | 0 | 0.198 | 0 | 0 | 0 | 0 | 0 |
|  | Brucellaceae | 0.019 | 0 | 0 | 0 | 0 | 0 | 0 |
|  | Caulobacteraceae | 0 | 0.89 | 0 | 0 | 0.135 | 0 | 0.026 |
|  | Erythrobacteraceae | 0 | 0 | 0.015 | 0 | 0 | 0 | 0 |
|  | Reyranella | 0 | 0.021 | 0 | 0 | 0 | 0 | 0 |
|  | Rhizobiaceae | 0.030 | 0 | 0.042 | 0 | 0 | 0 | 0 |
|  | Rhodospirillaceae | 0 | 0.012 | 0.023 | 0 | 0.052 | 0.017 | 0 |
|  | Sphingomonadaceae | 0 | 0.027 | 0 | 0.024 | 0 | 0 | 0 |
| β-class | Comamonadaceae | 0 | 0 | 0.087 | 0.011 | 0.020 | 0.022 | 0.043 |
|  | Gallionellaceae | 0 | 0 | 0 | 0.031 | 0 | 0 | 0 |
|  | Oxalobacteraceae | 0 | 0 | 0 | 0 | 0 | 0 | 0.010 |
|  | Rhodocyclaceae | 0 | 0.047 | 0.255 | 0.028 | 0.012 | 0.039 | 0.039 |
| δ-class | Desulfobulbaceae | 0 | 0 | 0.023 | 0 | 0 | 0 | 0 |
|  | Polyangiaceae | 0 | 0 | 0 | 0.026 | 0 | 0 | 0 |
|  | Syntrophaceae | 0 | 0 | 0 | 0.013 | 0 | 0 | 0 |
|  | Syntrophorhabdus | 0 | 0 | 0 | 0.024 | 0 | 0 | 0 |
| γ-class | Enterobacteriaceae | 0.007 | 0.160 | 0.038 | 0 | 0 | 0 | 0.013 |
|  | Moraxellaceae | 0 | 0.166 | 0 | 0 | 0.012 | 0 | 0 |
|  | Xanthomonadaceae | 0.011 | 0 | 0.057 | 0.102 | 4.264 | 2.820 | 2.464 |
|  | Xanthomonadales/  Sinobacteraceae | 0 | 0 | 0 | 0.006 | 0 | 0 | 0 |
