# Supplementary material for: Azolla filiculoides L. as a source of metal-tolerant microorganisms
Source: PLoS One. 2020 May 6;15(5):e0232699. doi: 10.1371/journal.pone.0232699 (PMC7202617; doi:10.1371/journal.pone.0232699)
Supplement: S6 Table — (DOCX) [file pone.0232699.s006.docx]

**S6 Table. The composition of ‘Other’ cluster for each treatment presented as relative abundance (%) of the phylum Bacteroidetes.**

| **Genus** | **treatment** | | | | | | |  |
| --- | --- | --- | --- | --- | --- | --- | --- | --- |
|  | **control** | **+Pb** | **+Cd** | **+Cr(VI)** | **+Ni** | **+Au** | **+Ag** | |
| *Ferruginibacter* | 0 | 5.668 | 0 | 2.041 | 0 | 0 | 0 | |
| *Vitellibacter* | 0 | 0 | 0 | 6.122 | 0 | 0 | 0 | |
| *Prevotella* | 0 | 0 | 2.030 | 2.551 | 0 | 0 | 0 | |
| *Pedobacter* | 0 | 0 | 0 | 3.061 | 0 | 0 | 0 | |
| *Rhodocytophaga* | 0 | 2.024 | 0 | 0 | 0 | 0.182 | 0 | |
| *Bacteroides* | 0 | 1.619 | 0 | 0 | 0 | 0 | 0 | |
| *Hymenobacter* | 0 | 0 | 0.923 | 0 | 0 | 0 | 0 | |
| *Spirosoma* | 0 | 0 | 0.738 | 0 | 0 | 0 | 0 | |
| *Flavisolibacter* | 0 | 0 | 0.554 | 0 | 0 | 0 | 0 | |
| *Alistipes* | 0 | 0 | 0.369 | 0 | 0 | 0 | 0 | |
