# Supplementary material for: Azolla filiculoides L. as a source of metal-tolerant microorganisms
Source: PLoS One. 2020 May 6;15(5):e0232699. doi: 10.1371/journal.pone.0232699 (PMC7202617; doi:10.1371/journal.pone.0232699)
Supplement: S7 Table — (DOCX) [file pone.0232699.s007.docx]

**S7 Table. The relative abundance (%) of unidentified microorganisms (percentage of a given phylum) for each treatment and their assignment to corresponding family.**

| **Phylum** | **Family** | **treatment** | | | | | | |
| --- | --- | --- | --- | --- | --- | --- | --- | --- |
|  |  | **control** | **+Pb** | **+Cd** | **+Cr(VI)** | **+Ni** | **+Au** | **+Ag** |
| Bacteroidetes | Chitinophagaceae | 0 | 12.955 | 19.188 | 16.837 | 23.256 | 0.273 | 11.194 |
|  | Porphyromonadaceae | 0 | 0 | 0 | 5.612 | 0 | 0 | 0 |
|  | Sphingobacteriaceae | 0 | 5.263 | 0 | 0 | 0 | 0 | 0 |
|  | Ohtaekwangia | 0 | 0 | 3.875 | 0 | 0 | 0 | 0 |
|  | Flavobacteriaceae | 0 | 1.215 | 1.661 | 0 | 0 | 0 | 0 |
|  | Prolixibacteracea | 0 | 0 | 0 | 2.551 | 0 | 0 | 0 |
|  | Cytophagaceae | 0 | 0 | 1.661 | 0 | 0 | 0 | 0 |
|  | Chryseolinea | 0 | 1.619 | 0 | 0 | 0 | 0 | 0 |
| Firmicutes | Veillonellaceae | 5.839 | 12.691 | 1.645 | 14.954 | 16.667 | 3.774 | 0 |
|  | Ruminococcaceae | 0 | 0 | 1.316 | 1.170 | 0 | 0 | 0 |
|  | Lachnospiraceae | 0 | 0 | 1.974 | 0 | 0 | 0 | 0 |
| Actinobacteria | Microbacteriaceae | 0 | 6.084 | 3.990 | 1.036 | 52.273 | 0 | 0 |
|  | Coriobacteriaceae | 0 | 0 | 1.746 | 0 | 0 | 0 | 6.452 |
|  | Thermomonosporaceae | 0 | 0 | 0 | 0 | 0 | 6.140 | 0 |
|  | Geodermatophilaceae | 0 | 0 | 0 | 0 | 0 | 5.263 | 0 |
|  | Iamiaceae | 0 | 0 | 1.496 | 0 | 0 | 2.632 | 0 |
|  | Promicromonosporaceae | 0 | 2.662 | 0 | 0 | 0 | 0 | 0 |
|  | Micromonosporaceae | 0 | 0 | 1.247 | 0.888 | 0 | 0 | 0 |
|  | Micrococcaceae | 0 | 0 | 1.496 | 0 | 0 | 0 | 0 |
|  | Nocardioidaceae | 0 | 0 | 0 | 0.444 | 0 | 0 | 0 |
