# Supplementary material for: Azolla filiculoides L. as a source of metal-tolerant microorganisms
Source: PLoS One. 2020 May 6;15(5):e0232699. doi: 10.1371/journal.pone.0232699 (PMC7202617; doi:10.1371/journal.pone.0232699)
Supplement: S8 Table — (DOCX) [file pone.0232699.s008.docx]

**S8 Table. The composition of ‘Oher’ cluster for each treatment presented as relative abundance (%) of the phylum Firmicutes.**

| Genera | treatment | | | | | | | |
| --- | --- | --- | --- | --- | --- | --- | --- | --- |
|  | control | +Pb | +Cd | +Cr(VI) | +Ni | +Au | +Ag |  |
| *Listeria* | 0 | 0 | 4.605 | 1.040 | 0 | 9.434 | 0 |  |
| *Romboutsia* | 3.650 | 5.033 | 0.000 | 3.381 | 0 | 0 | 0 |  |
| *Finegoldia* | 5.109 | 0 | 3.618 | 0 | 0 | 0 | 0 |  |
| *Peptoniphilus* | 0 | 0 | 1.645 | 0 | 6.667 | 0 | 0 |  |
| *Lactococcus* | 0 | 1.313 | 2.303 | 4.421 | 0 | 0 | 0 |  |
| *Anaerococcus* | 2.190 | 0 | 0.000 | 0 | 0 | 0 | 3.030 |  |
| *Brochothrix* | 3.650 | 1.313 | 0.000 | 0 | 0 | 0 | 0 |  |
| *Granulicatella* | 0 | 1.532 | 1.974 | 0.910 | 0 | 0 | 0 |  |
| *Peptostreptococcus* | 3.650 | 0 | 0.000 | 0 | 0 | 0 | 0 |  |
| *Exiguobacterium* | 0 | 0 | 3.618 | 0 | 0 | 0 | 0 |  |
| *Sporosarcina* | 0 | 0 | 0.000 | 2.601 | 0 | 0 | 0 |  |
| *Veillonella* | 0 | 0 | 1.974 | 0.520 | 0 | 0 | 0 |  |
| *Tumebacillus* | 0 | 0 | 1.974 | 0 | 0 | 0 | 0 |  |
| *Roseburia* | 0 | 0 | 1.645 | 0 | 0 | 0 | 0 |  |
| *Leuconostoc* | 0 | 0 | 1.316 | 0 | 0 | 0 | 0 |  |
| *Dialister* | 0 | 0 | 0 | 0.910 | 0 | 0 | 0 |  |
| *Clostridium_XlVa* | 0 | 0.875 | 0 | 0 | 0 | 0 | 0 |  |
| *Lachnoanaerobaculum* | 0 | 0 | 0 | 0.780 | 0 | 0 | 0 |  |
| *Lactobacillus* | 0 | 0 | 0 | 0.650 | 0 | 0 | 0 |  |
| *Paenibacillus* | 0 | 0 | 0 | 0.390 | 0 | 0 | 0 |  |
| *Ruminococcus* | 0 | 0 | 0 | 0.390 | 0 | 0 | 0 |  |
| *Dorea* | 0 | 0 | 0 | 0.260 | 0 | 0 | 0 |  |
