# Supplementary material for: Azolla filiculoides L. as a source of metal-tolerant microorganisms
Source: PLoS One. 2020 May 6;15(5):e0232699. doi: 10.1371/journal.pone.0232699 (PMC7202617; doi:10.1371/journal.pone.0232699)
Supplement: S9 Table — (DOCX) [file pone.0232699.s009.docx]

**S9 Table. The composition of ‘Other’ cluster for each treatment presented as relative abundance (%) of the phylum Actinobacteria.**

| **Genus** | **treatment** | | | | | | | |
| --- | --- | --- | --- | --- | --- | --- | --- | --- |
|  | **control** | **+Pb** | **+Cd** | **+Cr(VI)** | **+Ni** | **+Au** | **+Ag** |  |
| *Nocardioides* | 0 | 3.422 | 4.988 | 4.142 | 0 | 1.754 | 9.677 |  |
| *Pseudonocardia* | 0 | 2.281 | 0 | 8.728 | 0 | 0 | 0 |  |
| *Nocardia* | 0 | 0 | 0 | 0 | 0 | 0 | 9.677 |  |
| *Leifsonia* | 0 | 0 | 0 | 0 | 0 | 8.772 | 0 |  |
| *Aeromicrobium* | 0 | 3.422 | 0.748 | 3.846 | 0 | 0 | 0 |  |
| *Janibacter* | 0 | 0 | 7.980 | 0 | 0 | 0 | 0 |  |
| *Gaiella* | 0 | 4.183 | 0.998 | 0.740 | 0 | 0 | 0 |  |
| *Arthrobacter* | 0 | 2.662 | 2.244 | 0 | 0 | 0 | 0 |  |
| *Actinomyces* | 0 | 3.422 | 0 | 1.036 | 0 | 0 | 0 |  |
| *Blastococcus* | 0 | 2.662 | 0 | 1.183 | 0 | 0 | 0 |  |
| *Ilumatobacter* | 0 | 1.901 | 0.748 | 1.183 | 0 | 0 | 0 |  |
| *Kribbella* | 0 | 0 | 2.993 | 0 | 0 | 0 | 0 |  |
| *Actinomadura* | 0 | 0 | 2.993 | 0 | 0 | 0 | 0 |  |
| *Dactylosporangium* | 0 | 2.662 | 0 | 0 | 0 | 0 | 0 |  |
| *Herbidospora* | 0 | 2.662 | 0 | 0 | 0 | 0 | 0 |  |
| *Rothia* | 0 | 0 | 2.494 | 0 | 0 | 0 | 0 |  |
| *Brevibacterium* | 0 | 1.141 | 0 | 1.183 | 0 | 0 | 0 |  |
| *Brachybacterium* | 0 | 2.281 | 0 | 0 | 0 | 0 | 0 |  |
| *Modestobacter* | 0 | 0 | 1.995 | 0 | 0 | 0 | 0 |  |
| *Nakamurella* | 0 | 1.901 | 0 | 0 | 0 | 0 | 0 |  |
| *Rhodococcus* | 0 | 0 | 0.998 | 0.888 | 0 | 0 | 0 |  |
| *Streptosporangium* | 0 | 0 | 0 | 1.479 | 0 | 0 | 0 |  |
| *Microlunatus* | 0 | 0 | 0 | 1.183 | 0 | 0 | 0 |  |
| *Actinocatenispora* | 0 | 0 | 0.998 | 0 | 0 | 0 | 0 |  |
| *Thermoleophilum* | 0 | 0 | 0.499 | 0 | 0 | 0 | 0 |  |
| *Conexibacter* | 0 | 0 | 0 | 0.444 | 0 | 0 | 0 |  |
