# Supplementary material for: Azolla filiculoides L. as a source of metal-tolerant microorganisms
Source: PLoS One. 2020 May 6;15(5):e0232699. doi: 10.1371/journal.pone.0232699 (PMC7202617; doi:10.1371/journal.pone.0232699)
Supplement: Attachment — Submitted filename: Plos One Comments.docx [file pone.0232699.s010.docx]

**General comments:**

The present manuscript entitled " The endophytic microbiome of *Azolla filiculoides* L. exposed to selected heavy metals” contains a good idea and impressive amount of work to explain the tolerance ability of target fern (in combination with microbiome) exposed to selected heavy metals. This research is problem-based and might help scientists working on phytoremediation or bioremediation. Nevertheless, authors need some improvement in the manuscript for their possible acceptance in the Journal. Following are some suggestions to be incorporated before making any decision.

**Specific Comments:**

**Abstract section:**

Make clear hypothesis at the start of this section.

Overall it is fine

**Introduction section:**

In line 42-43: Rephrase the sentence. Also, delete the extra addition of “and” and which” from these lines.

In line 47-48: Delete extra commas (,)

In line 53-56: Rewrite the sentence to make it clearer. Avoid repetition of words

In line 72: “One plant” should be “Among aquatic ferns” or “Among hyperaccumulators” and “heavy metal phytoremediation” should be “heavy metal removal by phytoremediation”, change accordingly.

In line 75: Mention the particular heavy metals

In line 77-78: Clearly write the sentence i.e. “their………microorganisms”

It is suggested to add few sentences describing the phytotoxic impacts of selected heavy metals and their removal by phytoremediation strategies.

Further improve the study objectives at the end of this section.

**Materials and methods section:**

In line 98: The selection of particular heavy metals and their doses are not clearly described. Please explain them within the text or provide reference (not unpublished data).

In line 102: The collection of plant material was carried out after one week, specify the reason?

In line 149-151: Re-arrange the sentence

**Results section:**

In line 185 and so on: Please cite all figure numbers inside the text (not with headings).

In line 244: Delete either “percentage” or “%”.

In line 247: Use “%” with last number/value only.

In line 267: First use “lead (Pb)” and then “Pb” (also for other metals) throughout the section. Be consistent

In line 352: Correct as “_―_gp3”

Explanation of results is fine.

**Discussion section:**

In line 407: “A. filiculoides” should be Italic and write genus “Azolla” due to first appearance.

In line 410: “Only Pb, Cd and Cr resulted in higher abundances of taxa”, Please check “Ag” also showing significant abundance such as in figure 1.

In line 465-466: Re-write the sentence “In 1983…………microorganisms”

In line 507:” which” should be replaced with “whose” and “was the highest” should be “was highest”. Please re-check in other sections

Authors are suggested to further discuss this section with more possible mechanistic approaches. There are some related articles which can help the authors to understand the similar topic; *Biologia Plantarum*, 2014, *58*(1), 131-138; *Ecotoxicology and Environmental Safety*, 2014, *102*, 25-33; and *Plant Physiology and Biochemistry*, 2019, *145*, 142-152.

**Conclusion section:**

At the end of this section, it is suggested to add one or two more sentences concerning the future prospects of the present study.

Overall, there are some grammatical errors throughout the manuscript. All sections of the present study were well enough explained and discussed (although need correction/improvement).
